# An investigation of the effects of the antioxidants, ebselen or N-acetyl cysteine on human peripheral blood mononuclear cells and T cells

**DOI:** 10.1186/1742-4933-10-7

**Published:** 2013-02-21

**Authors:** Shiva Marthandan, Paul Hyland, Graham Pawelec, Yvonne Barnett

**Affiliations:** 1 Leibniz Institute for Age Research, Jen Age (Jena Centre for Systems Biology of Ageing), Beutenbergstrasse 11, Jena, D-07745, Germany; 2 National Blood Authority, Canberra, ACT 2601, Australia; 3 Tübingen Ageing and Tumour Immunology Group, Center for Medical Research, University of Tübingen Clinical School, Waldhörnlestr. 22, Tübingen, D-72072, Germany; 4 School of Science and Technology, College of Arts and Science, Nottingham Trent University, Clifton Lane, Nottingham, England, NG11 8NS, UK

**Keywords:** Ebselen, NAC, PBMCs, Proliferative capacity, Lifespan, DNA damage, GSH: GSSG ratio, Total glutathione

## Abstract

**Background:**

The research literature has documented age-related increases in genetic damage, including oxidative DNA damage, in human T lymphocytes, *in vitro* and *ex vivo*. Such damage has the potential to interfere with the ability of the T cells to proliferate at times when they need to, such as when antigen challenged. The consequence of this could be a sub-optimal immune response *in vivo.*

**Context and purpose:**

The purpose of the research reported in this paper was to investigate the impact of two antioxidants, which can be administered *in vivo,* Ebselen and N-acetyl L-cysteine, on the age-related increase in genetic damage, and on T cell proliferation and lifespan. *In vitro* human T cell clones, *ex vivo* peripheral blood mononuclear cells or T cells were supplemented with different concentrations of antioxidants, under standard conditions and for different periods of time. A range of assays were then applied in order to determine any impact of the antioxidants.

**Results:**

30 μM ebselen or 7.5 mM N-acetyl L-cysteine supplementation resulted in a significantly higher intracellular GSH: GSSG ratio. This increased ratio was accompanied by reduced levels of oxidative DNA damage in established CD4^+^ human T cell clones, from a young or a middle-aged donor. Additionally, cultures of primary human peripheral blood mononuclear cells and CD4^+^ T cells from donors aged 25–30 or 55–60 years were also supplemented with these agents. Cells from all sources exhibited increased proliferation, and in the case of the T cell clones, an increase in cumulative population doublings. Neither ebselen nor N-acetyl L-cysteine had such effects on clones supplemented from the midpoint of their *in vitro* lifespan.

**Conclusions:**

Ebselen and N-acetyl L-cysteine, under certain conditions, may have anti-immunosenescent potential in T cells in *in vitro* clonal and *ex vivo* polyclonal culture models.

## Background

Previous work has revealed an age-associated increase in DNA damage in human peripheral blood-derived T cell clones (TCCs) *in vitro* under 20% O_2_ tension culture conditions [1]. DNA damage levels and mutation frequency have also been shown to be increased with age in polyclonal human lymphocytes [2]. T cell function is known to decline with age, associated with increased susceptibility to infection, cancer and a variety of diseases. Naive/memory T cells need to undergo rapid clonal expansion on contact with their specific antigen, for which they carry unique antigen receptors, to generate an effective immune response. DNA damage, which can result in cell cycle arrest and/or apoptosis, may impact on the effectiveness of an immune response, by interfering with clonal expansion.

Reactive oxygen species (ROS), from a wide variety of intrinsic and extrinsic sources, are a major cause of DNA damage *in vivo*[3]. ROS have been implicated in several human degenerative diseases of ageing [4]. T cells are also exposed to high concentrations of ROS and reactive nitrogen intermediates, produced as a normal consequence of an immune response, at sites of inflammation. *In vivo* levels of antioxidants and DNA repair mechanisms represent two types of T cell defence systems that can counteract ROS-induced DNA damage [5]. An age-related decline of DNA repair capacities has been previously reported in CD4^+^ TCCs cultured *in vitro* in 20% O_2_ tension [6,7]. Here, we set out to determine the potential of the antioxidants 2-phenyl-1,2-benzisoselenazol-3 (2H)-one (ebselen) and N-acetyl L-cysteine (NAC) to protect T cells from the damaging effects of ROS.

Ebselen is a synthetic, lipid soluble seleno-organic compound having potent antioxidant capacity. It is also a novel anti-inflammatory agent having glutathione-peroxidase like activity, first described in 1984 [8]. NAC is an antioxidant containing an acetylated form of the amino acid L-cysteine that functions as a precursor of glutathione synthesis. Glutathione is an important thiol involved in cellular detoxification [9]. The presence of sulfhydryl groups in NAC also enables the neutralisation of free radicals. Previous publications have reported on the ability of ebselen to protect HepG_2_ cells [8], human HL-60 [10] and PC-12 cells [11] against H_2_O_2_ induced cell death and DNA damage.

This investigation revealed that certain concentrations of these antioxidants resulted in significant ROS scavenging in T cell samples *ex vivo* irrespective of the age group of the donors, but in TCCs, only when supplemented from a young *in vitro* age.

## Results

### The impact of ebselen or NAC on *in vitro* proliferative capacity and lifespan of CD4^+^ TCCs and proliferation capacity of human PBMCs and CD4^+^ T cells *ex vivo*

The effect of different concentrations of ebselen or NAC supplementation, from an early *in vitro* age (34.5 and 31.0 PD), on the proliferative capacity and *in vitro* lifespan of the 400–23 and 385–7 TCCs was investigated. The results in Table 1 show that supplementation of either 400–23 or 385–7 TCCs with 30 μM ebselen, 5 or 7.5 mM NAC, from a young *in vitro* age, resulted in a higher number of PDs per week and a significant increase in lifespan (cumulative PD; not significant following 5 mM NAC) of the TCCs, compared to non-supplemented clones. Neither 10 μM ebselen nor 1.25 mM NAC had any significant impact on the average number of PDs/week or the lifespan of CD4^+^ TCCs derived from donors of either age group, compared to non-supplemented clones.

**Table 1:** **Impact of 30 μM ebselen or 7.5 mM NAC on proliferative capacity and lifespan of TCC supplemented from a young *in vitro *age**

**Clone (Age of the donor)**	**Initial PD**	**Concentration**	**Average PD per week**	**Cumulative PD achieved at the end of lifespan in culture**
400-23 (26 year old donor)	34.5	Control	0.7	44.9
400-23 (26 year old donor)	34.5	10 μM ebselen	0.8	46.9
400-23 (26 year old donor)	34.5	30 μM ebselen	1.2	56.2*
400-23 (26 year old donor)	34.5	60 μM ebselen	Inhibited growth after one week in culture	34.5
400-23 (26 year old donor)	34.5	80 μM ebselen	Inhibited growth after one week in culture	34.5
400-23 (26 year old donor)	34.5	100 μM ebselen	Inhibited growth after one week in culture	34.5
400-23 (26 year old donor)	34.5	Control	0.9	49.1
400-23 (26 year old donor)	34.5	1.25 mM NAC	0.8	48.4
400-23 (26 year old donor)	34.5	5 mM NAC	1.1	53.5
400-23 (26 year old donor)	34.5	7.5 mM NAC	1.2	56.1*
400-23 (26 year old donor)	34.5	10 mM NAC	Inhibited growth after two weeks in culture	35.8
385-7 (45 year old donor)	31.0	Control	0.9	44.4
385-7 (45 year old donor)	31.0	10 μM ebselen	0.9	44.9
385-7 (45 year old donor)	31.0	30 μM ebselen	1.4	50.8*
385-7 (45 year old donor)	31.0	60 μM ebselen	Inhibited growth after one week in culture	31.0
385-7 (45 year old donor)	31.0	80 μM ebselen	Inhibited growth after one week in culture	31.0
385-7 (45 year old donor)	31.0	100 μM ebselen	Inhibited growth after one week in culture	31.0
385-7 (45 year old donor)	31.0	Control	1.0	47.2
385-7 (45 year old donor)	31.0	1.25 mM NAC	1.0	48.7
385-7 (45 year old donor)	31.0	5 mM NAC	1.3	52.8
385-7 (45 year old donor)	31.0	7.5 mM NAC	1.4	55.8*
385-7 (45 year old donor)	31.0	10 mM NAC	Inhibited growth after one week in culture	31.0

Values statistically different from their controls (Student’s *t*-test, 95% confidence level) are indicated with an asterisk. * p < 0.05, Significantly higher than the proliferative capacity of non-supplemented cells.

When the TCCs were supplemented from later points in their *in vitro* lifespan (58.7 and 63.4 PD) the analyses revealed marginal or no increase in proliferative capacity or lifespan, compared to control clones. Higher concentrations of ebselen (60-100 μM) or NAC (10 mM) inhibited the growth of CD4^+^ TCCs derived from either age group of donors (Table 1).

**Figure 1:**
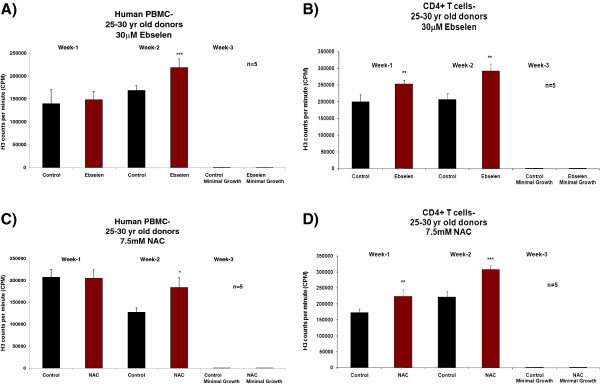
**The impact of 30 μM ebselen or 7.5 mM NAC on the proliferation capacity in human PBMCs or CD4**^**+ **^**T cells *ex vivo *isolated from fresh human blood derived from five different healthy donors, in the age group of 25–30 years supplemented *ex vivo *for three weeks. A** &**C** denotes the impact in human PBMCs and **B** &**D** denotes the impact on CD4^+^ T cells. The bars indicate the mean ± S.D. Values statistically different from their controls (Student’s *t*-test, 95% confidence level) are indicated with an asterisk. * p < 0.05, ** p < 0.01, *** p < 0.001 - Significantly higher than the proliferative capacity of non-supplemented cells.

**Figure 2:**
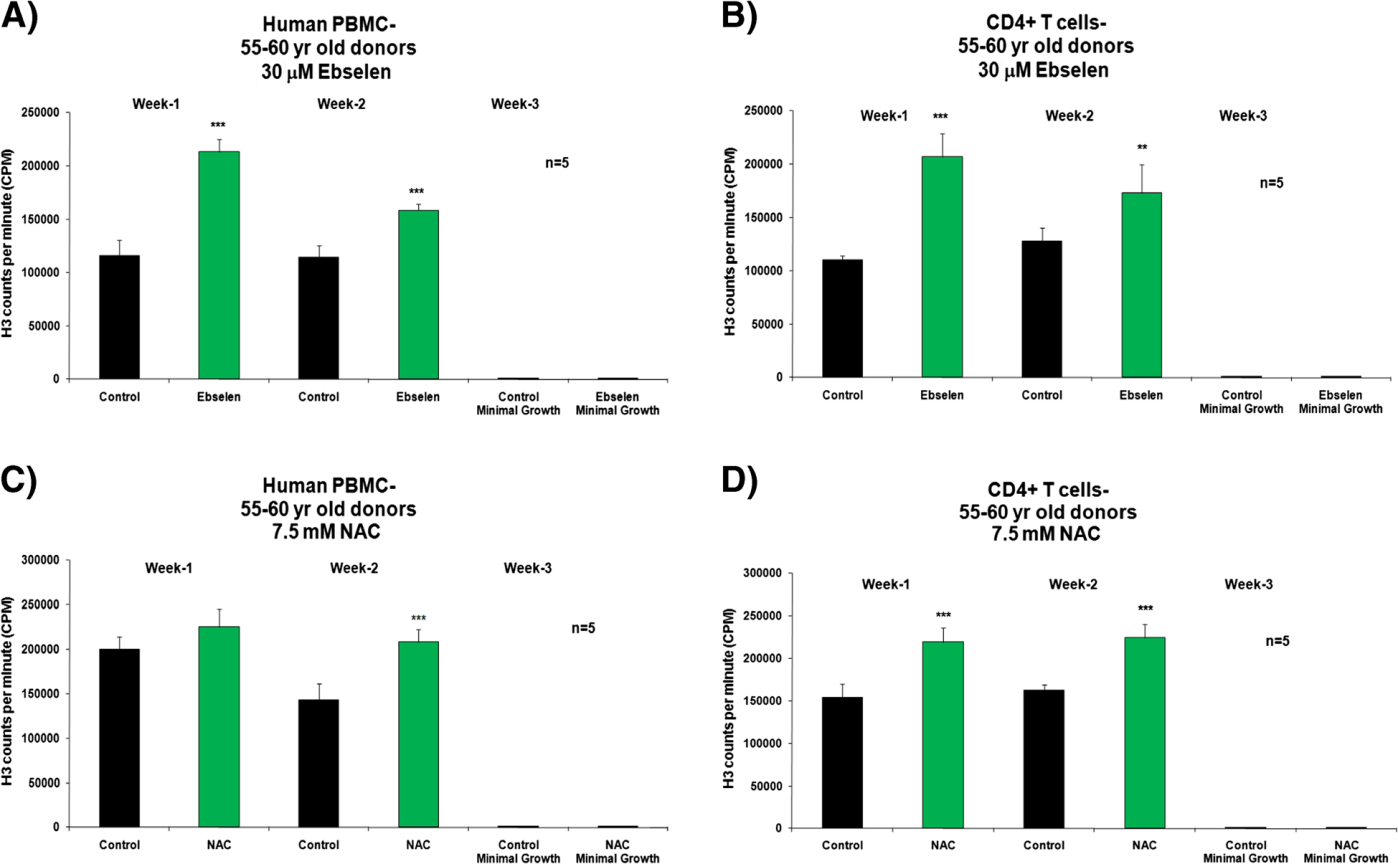
**The effect *ex vivo *supplementation for three weeks with 30 μM ebselen or 7.5 mM NAC on the proliferation capacity of human PBMCs (A and C) or CD4**^**+ **^**T cells *ex vivo *isolated from fresh human blood derived from five different healthy donors aged 55–60 years. A** &**B** denotes the effect of 30 μM ebselen and **C** &**D** denotes the effect of 7.5 mM NAC. The bars indicate the mean ± S.D. Values statistically different from their controls (Student’s *t*-test, 95% confidence level) are indicated with an asterisk. ** p < 0.01, *** p < 0.001 - Significantly higher than the proliferative capacity of non-supplemented cells.

Figure 1, A and C, shows a significant increase (Student’s *t*-test, 95% confidence levels) in the proliferative capacity of human PBMCs from donors in the 25–30 years age group after the second week of supplementation *ex vivo* with 30 μM ebselen or 7.5 mM NAC, compared to non-supplemented cells. In experiments with separated CD4^+^ T cells, there was an increase in proliferative capacity, which reached statistical significance at both 1 and 2 weeks of supplementation with 30 μM ebselen or 7.5 mM NAC (Figure 1, B and D). Experiments on the PBMCs and CD4^+^ T cells from donors aged 55–60 years revealed that 30 μM ebselen or 7.5 mM NAC significantly increased (Student’s *t*-test, 95% confidence levels) the proliferative capacity (Figure 2A-D). 30 μM ebselen (Figure 2A and B) significantly increased the proliferative capacity after both 1 and 2 weeks of supplementation in human PBMCs and CD4^+^ T cells. 7.5 mM NAC did so only after the second week of supplementation (Figure 2C). In CD4^+^ T cells (Figure 2D), 7.5 mM NAC supplementation resulted in a significant increase in proliferative capacity after both 1 and 2 weeks of supplementation. The results obtained following supplementation of the PBMCs or CD4^+^ T cells (from either age group of donors) *ex vivo* with 5 mM NAC were not significantly different to the results obtained for 7.5 mM NAC. The impact of each antioxidant on primary cultures of human PBMCs and CD4^+^ T cells, from either donor age group, was not significantly different. The number of live cells was too low after three weeks of culture of either PBMCs or CD4^+^ T cells *ex vivo* to carry out any analysis. Furthermore, the proliferative capacity observed for the PBMCs or CD4^+^ T cells derived from each of the five different donors in each of the above cases was not significantly different during the three week *ex vivo* culture period.

### The impact of ebselen or NAC on redox status and total glutathione levels in human CD4^+^ TCCs *in vitro*, PBMCs and CD4^+^ T cells *ex vivo*

**Figure 3:**
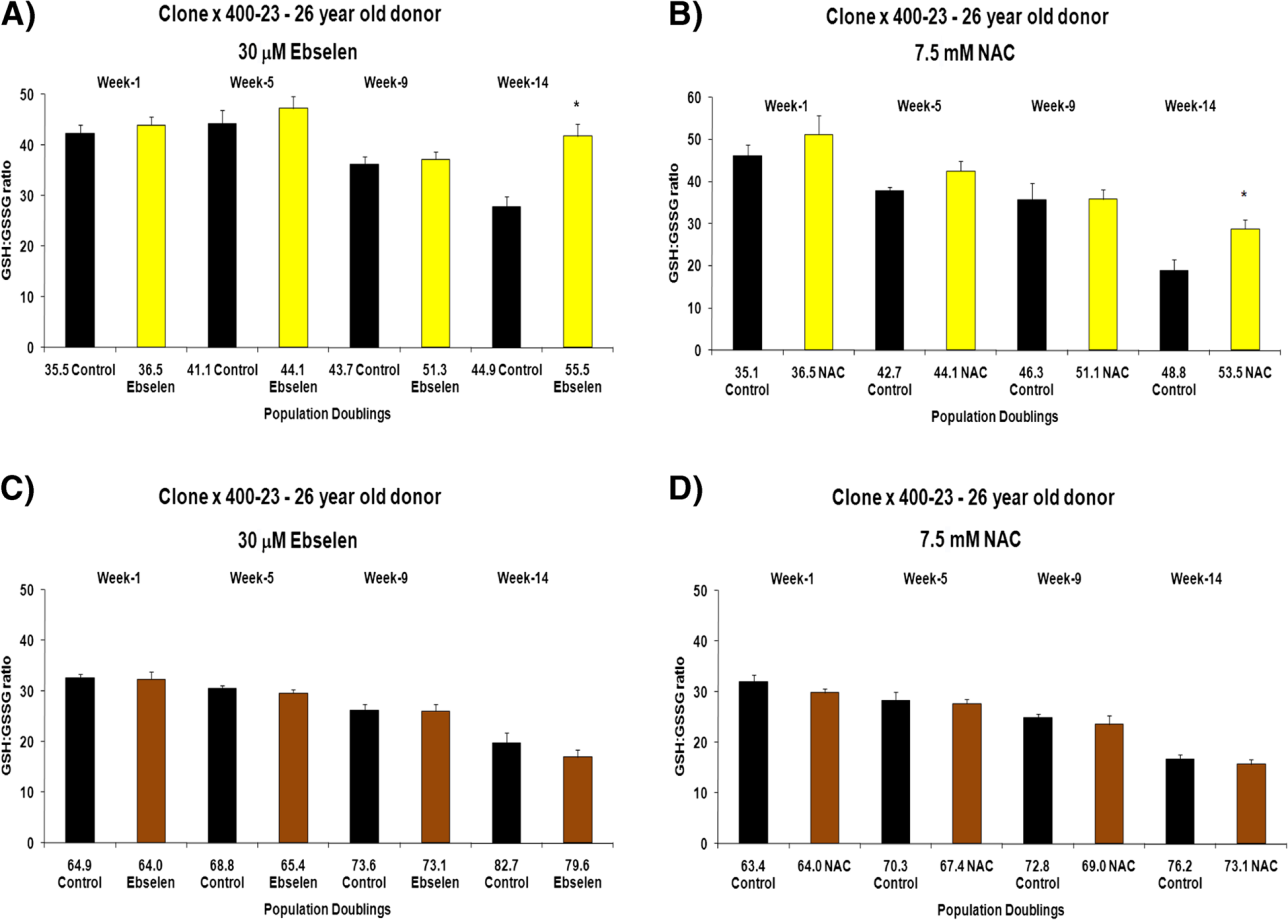
**The effect of 30 μM ebselen or 7.5 mM NAC on intracellular GSH: GSSG ratio in the human CD4**^**+ **^**TCC 400–23 when supplemented from a young *in vitro *age or from a later time point in the *in vitro *lifespan. A** &**B** denotes the impact of either antioxidant supplemented from a young *in vitro* age and **C** &**D** denotes the impact of these antioxidants when supplemented from the later stages of their *in vitro* lifespan. The bars indicate the mean ± S.D. Values statistically different from their controls (Student’s *t*-test, 95% confidence level) are indicated with an asterisk. * p < 0.05 - Significantly higher than the ratio of non-supplemented cells.

**Figure 4:**
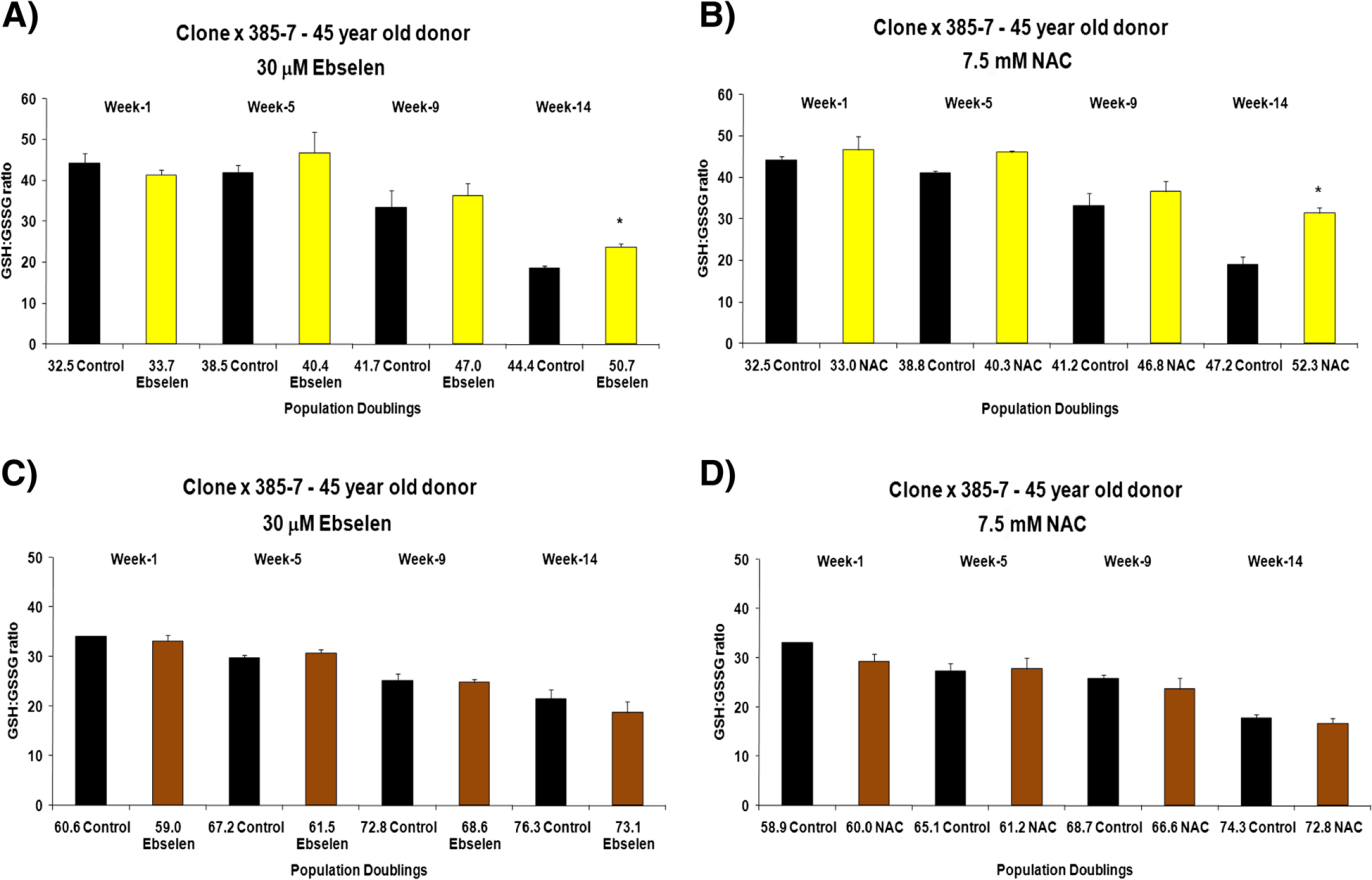
**The effect of 30 μM ebselen or 7.5 mM NAC on intracellular GSH: GSSG ratio in the human CD4**^**+ **^**TCC 385–7 when supplemented from a young *in vitro *age or from a later time point in the *in vitro *lifespan. A** &**B** denotes the impact of either antioxidant supplemented from a young *in vitro* age and **C** &**D** denotes the impact of these antioxidants when supplemented from the later stages of their *in vitro* lifespan. The bars indicate the mean ± S.D. Values statistically different from their controls (Student’s *t*-test, 95% confidence level) are indicated with an asterisk. * p < 0.05 – Significantly higher than the ratio of non-supplemented cells.

The effects of different concentrations of ebselen or NAC on intracellular redox status (GSH: GSSG ratio) and total glutathione levels in long-term cultured TCC and primary cultures of PBMCs and CD4^+^ T cells was determined at various time points. Figure 3 (A and B) shows the effects of supplementing the TCC 400–23, from an early *in vitro* age, with 30 μM ebselen or 7.5 mM NAC on GSH: GSSG ratio. The GSH: GSSG ratio was determined at various time points (cumulative population doublings) during the *in vitro* lifespan of 400–23. In contrast, Figure 3 (C and D) shows the effects of supplementation, from a later stage in the *in vitro* lifespan, with 30 μM ebselen or 7.5 mM NAC on the GSH: GSSG ratio. Supplementation from a young *in vitro* age with 30 μM ebselen (Figure 3A) or 7.5 mM NAC (Figure 3B) resulted in a higher GSH: GSSG ratio, when compared to non-supplemented, that reached statistical significance after 14 weeks. A similar set of results was obtained in a clone derived from a middle aged donor (4 A and B). 30 μM ebselen (Figure 3C) or 7.5 mM NAC (Figure 3D) supplementation from later stages of the *in vitro* lifespan did not have any measurable impact on the GSH: GSSG ratio. A similar set of results was obtained in a TCC, derived from a 45 year old donor, when supplemented with ebselen or NAC from a later stage of its *in vitro* lifespan (Figure 4C and D). There were no quantitative significant differences in the GSH: GSSG ratio in TCCs derived from a young donor (400–23) compared to the ratio in clones derived from a middle aged donor (385–7 clones). Testing other doses of ebselen or NAC on GSH: GSSG ratio in TCCs revealed that neither 10 μM ebselen nor 1.25 mM NAC significantly altered the GSH: GSSG ratio.

**Figure 5:**
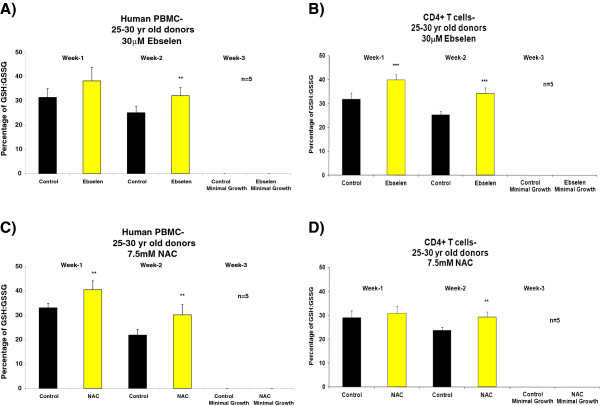
**The effect of three weeks *ex vivo *supplementation with 30 μM ebselen or 7.5 mM NAC on the intracellular GSH: GSSG ratio in human PBMCs or CD4**^**+ **^**T cells isolated from fresh human blood derived from five different healthy donors aged 25–30 years. A** &**B** denotes the effect of 30 μM ebselen and **C** &**D** denotes the effect of 7.5 mM NAC. The bars indicate the mean ± S.D. Values statistically different from their controls (Student’s *t*-test, 95% confidence level) are indicated with an asterisk. **p < 0.01, ***p <0.001 – Significantly higher than the ratio of non-supplemented cells.

**Figure 6:**
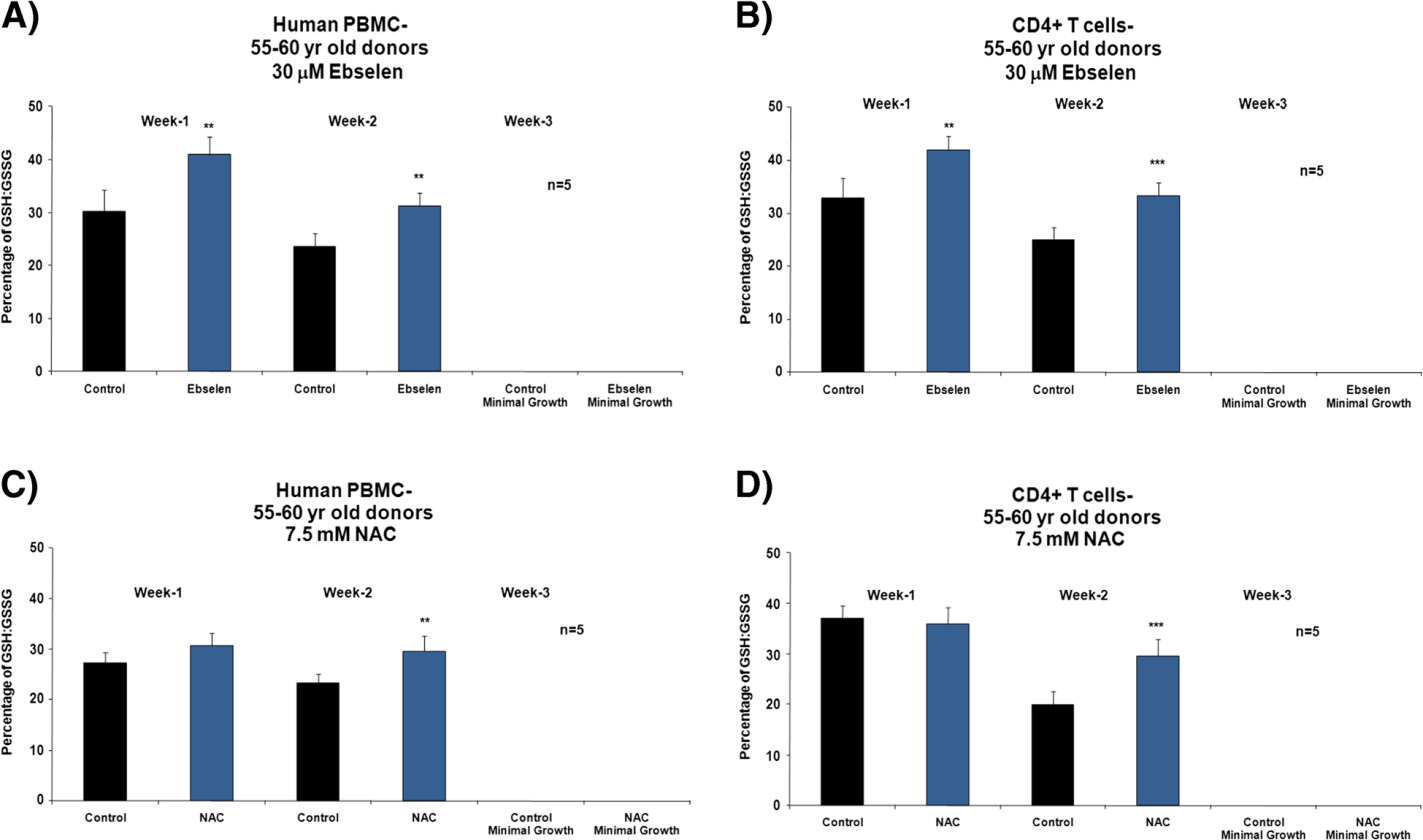
**The effect of three weeks *ex vivo *supplementation with 30 μM ebselen or 7.5 mM NAC on the intracellular GSH: GSSG ratio in human PBMCs or CD4**^**+ **^**T cells isolated from fresh human blood derived from five different healthy donors aged 55–60 years. A** &**C** denote the effect of antioxidants in human PBMCs and **B** &**D** denote the effect of antioxidants in CD4^+^ T cells. The bars indicate the mean ± S.D. Values statistically different from their controls (Student’s *t*-test, 95% confidence level) are indicated with an asterisk. **p < 0.01, ***p <0.001 – Significantly higher than the ratio of non-supplemented cells.

Figures 5 and 6 show the results of the effect of 30 μM ebselen (Figures 5A and B, 6A and B) or 7.5 mM NAC (Figures 5C and D, 6C and D) on the GSH: GSSG ratio at different culture time points of human PBMCs or CD4^+^ T cells derived from healthy 25–30 or 55–60 year olds. Figure 5A shows a significantly higher GSH: GSSG ratio in human PBMCs derived from 25–30 year old donors after two weeks of supplementation with 30 μM ebselen, compared to non-supplemented cells. This pattern was similar following 7.5 mM NAC supplementation of CD4^+^ T cells (Figure 5D). Figure 5 (B and C) shows a significantly higher GSH: GSSG ratio in CD4^+^ T cells and human PBMCs after one and two weeks of supplementation with 30 μM ebselen or 7.5 mM NAC, respectively, compared to non-supplemented cells. The results obtained for PBMCs or CD4^+^ T cells from 55–60 year old donors, also showed that 30 μM ebselen supplementation significantly increased the levels of GSH: GSSG ratio after one and two weeks of supplementation (Figure 6A and B). 7.5 mM NAC supplementation significantly increased the levels of GSH: GSSG ratio after 2 weeks (Figure 6C and D).

There were no significant differences between the GSH: GSSG ratio in cells from donors aged 25–30 years and the ratio in cells from donors aged 55–60 years. Also, there were no significant differences in the resultant GSH: GSSG ratios in PBMCs or CD4^+^ T cells supplemented with 5 mM NAC or 7.5 mM NAC (results not shown). Furthermore, individual GSH: GSSG ratios measured in human PBMCs and CD4^+^ T cells *ex vivo* derived from each of the five different donors in each of the above cases were not significantly different.

The results obtained for the measurement of total intracellular glutathione levels in the TCCs revealed that none of the concentrations of ebselen or NAC had any significant impact on total glutathione levels, compared to non-supplemented clones, either when supplemented from an early *in vitro* age or from a later point in the *in vitro* lifespan (results not shown). In contrast, 30 μM ebselen or 7.5 mM NAC supplementation significantly increased the total glutathione levels in primary cultures of PBMCs and CD4^+^ T cells from donors of either age group. 5 mM and 7.5 mM NAC supplementation had very similar effects on total intracellular glutathione levels. There were no significant differences between the total glutathione levels in cells from donors aged 25–30 years compared to cells from donors aged 55–60 years. Furthermore, the results did not reveal any differences in total glutathione levels between individual PBMCs and CD4^+^ T cell samples from each of the five different donors (results not shown).

### The impact of ebselen or NAC on levels of oxidative DNA damage in human CD4^+^ TCCs as a function of *in vitro* age, human PBMCs and CD4^+^ T cells *ex vivo*

**Figure 7:**
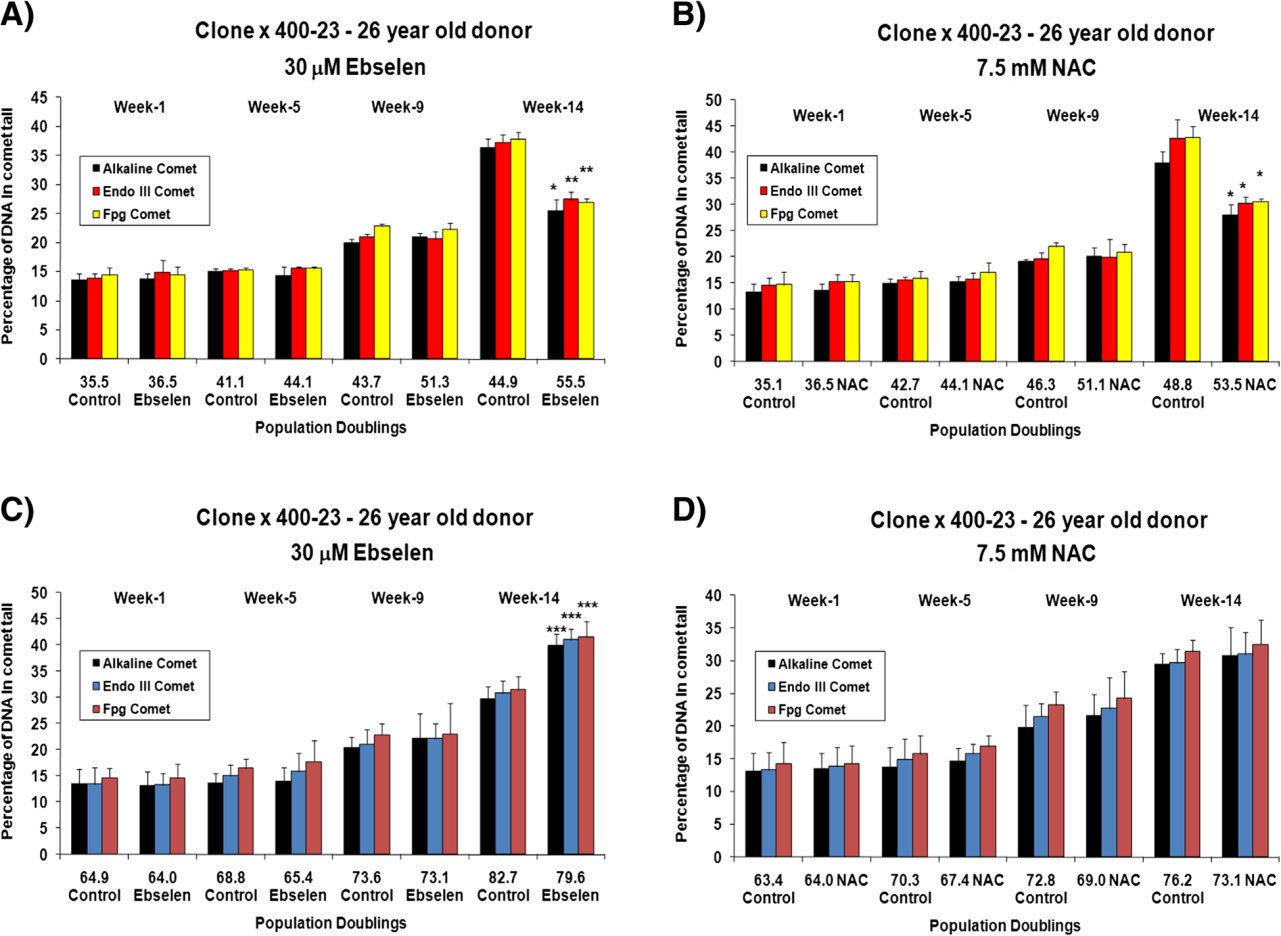
**The effect of 30 μM ebselen or 7.5 mM NAC on the levels of oxidative DNA damage in the 400–23 CD4**^**+ **^**TCC when supplemented from a young *in vitro *age or when supplemented from a later stage in the lifespan. A** &**C** illustrates the effect of 30 μM ebselen and **B** &**D** illustrates the effect of 7.5 mM NAC. The bars indicate the mean ± S.D. Values statistically different from their controls (Student’s *t*-test, 95% confidence level) are indicated with an asterisk. * p < 0.05, ** p < 0.01, *** p < 0.001 - Significantly higher than the levels of DNA damage in non-supplemented cells.

**Figure 8:**
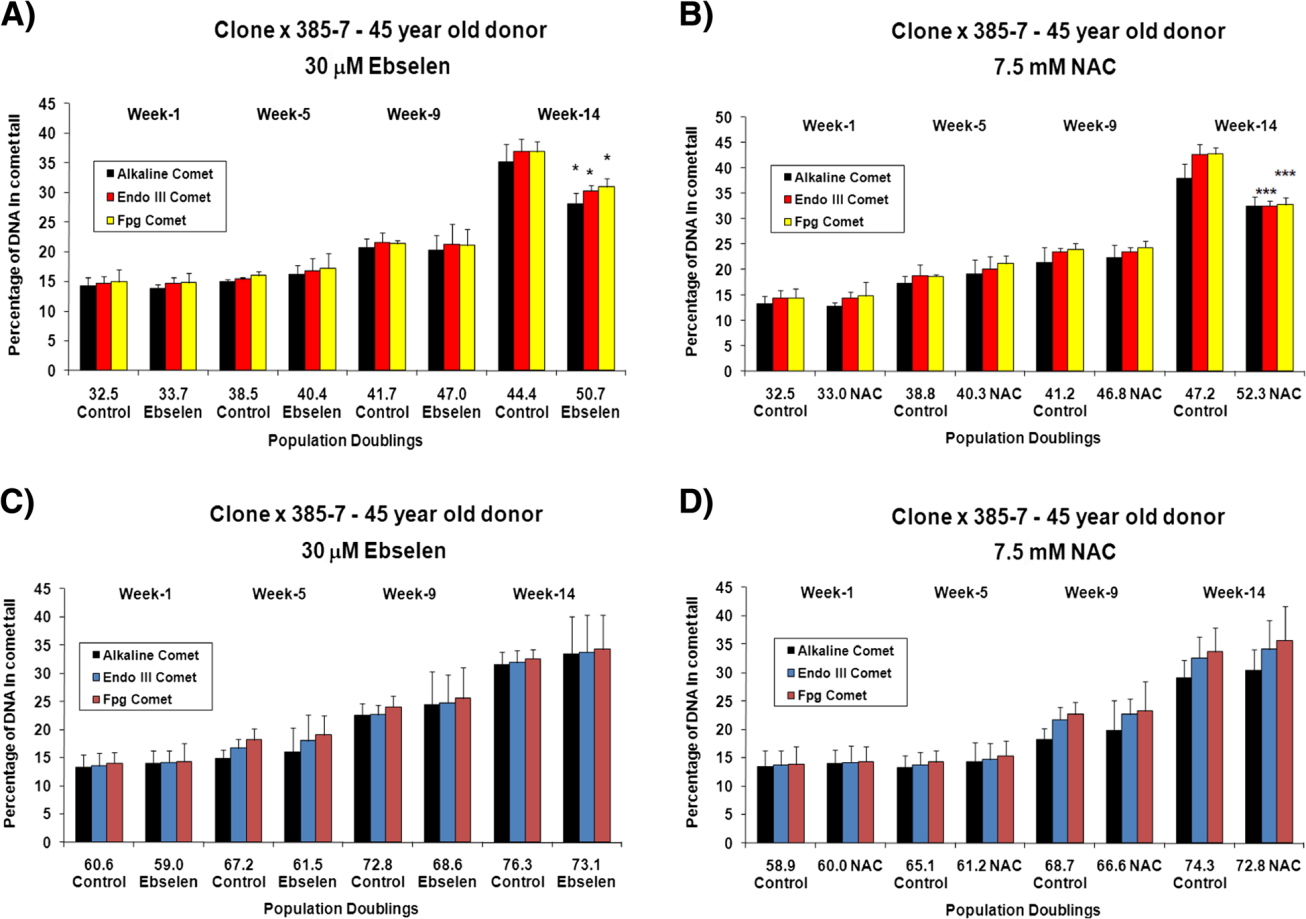
**The effect of 30 μM ebselen or 7.5 mM NAC on the levels of oxidative DNA damage in the 385–7 CD4**^**+ **^**TCC when supplemented from a young *in vitro *age or when supplemented from a later stage in the lifespan. A** &**C** illustrates the effect of 30 μM ebselen and **B** &**D** illustrates the effect of 7.5 mM NAC. The bars indicate the mean ± S.D. Values statistically different from their controls (Student’s *t*-test, 95% confidence level) are indicated with an asterisk. * p < 0.05, ** p < 0.01, *** p < 0.001 - Significantly higher than the levels of DNA damage in non-supplemented cells.

Aliquots of TCC samples were taken from culture at different time points and the effect of different concentrations of ebselen or NAC on the levels of oxidative DNA damage within the cells was determined using the Comet assay. In control samples, levels of oxidative damage to DNA increased as a function of *in vitro* age, in line with previously published findings [1,5]. Figures 7 and 8 (A and B) depicts the impact of 30 μM ebselen (7A and 8A) or 7.5 mM NAC (7B and 8B) on the levels of oxidative DNA damage in human CD4^+^ TCCs *in vitro* supplemented from a young *in vitro* age. The results show an increase in levels of oxidative DNA damage with time in culture, in both supplemented and non-supplemented clones. Supplemented clones had undergone more PDs after each sampled week of culture than non-supplemented clones when supplemented from a young *in vitro* age. When compared to non-supplemented clones, supplementation from a young *in vitro* age with 30 μM ebselen (7A and 8A) or 7.5 mM NAC (7B and 8B) did not result in any significant differences in the levels of oxidative DNA damage after one, five and nine weeks of culture, in the TCCs derived from either age group of donors. The reduction in levels of oxidative damage to DNA in supplemented clones reached statistical significance after fourteen weeks of culture.

The impact of other doses of ebselen or NAC on the levels of oxidative DNA damage was investigated. Neither 10 μM ebselen nor 5 mM NAC significantly altered the levels of oxidative DNA damage in CD4^+^ TCCs derived from a healthy 26 year old donor or a healthy 45 year old donor, compared to non-supplemented clones for any of the four time points investigated.

Figures 7 and 8 (C and D) show the impact of 30 μM ebselen (7C and 8C) or 7.5 mM NAC (7D and 8D) on the levels of oxidative DNA damage in human CD4^+^ TCCs *in vitro* supplemented from the later stages of their *in vitro* lifespan. In the case of TCC supplementation from the later stages of *in vitro* lifespan, no reductions in the levels of oxidative DNA damage, at the any of the time points examined, were found. Levels of oxidative DNA damage increased with increase of time in culture in both supplemented and non-supplemented clones. The non-supplemented clones underwent more PDs, compared to supplemented clones, at each of the weekly sampling points.

There were no significant differences in the levels of oxidative DNA damage, or in the extent of the impact of the antioxidants in TCCs derived from a young donor when compared with the cells from a middle aged donor (Figures 7 and 8 A, B, C and D).

**Figure 9:**
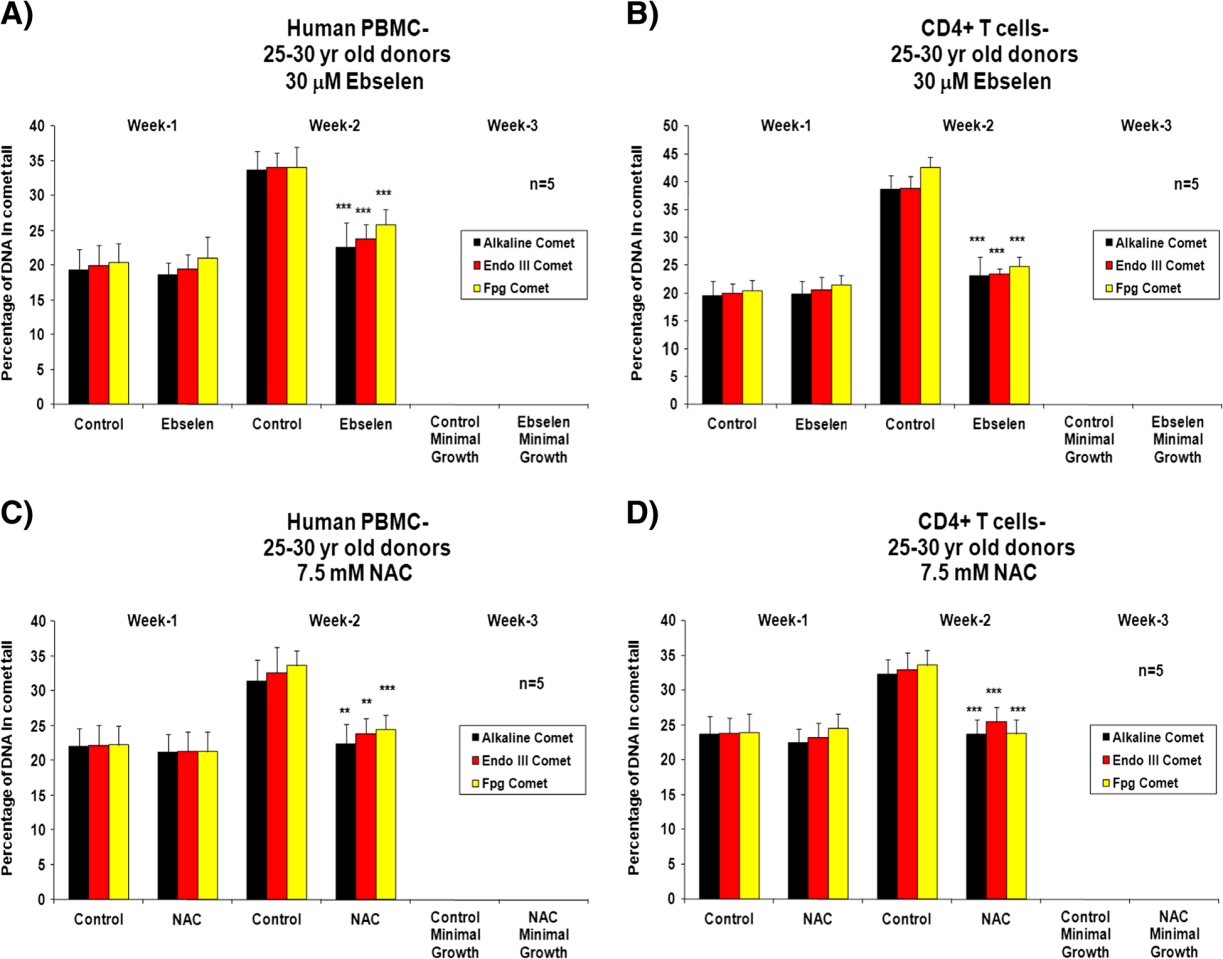
**The effect of three weeks *ex vivo *supplementation with 30 μM ebselen or 7.5 mM NAC on the levels of oxidative DNA damage in human PBMCs or CD4**^**+ **^**T cells *ex vivo *isolated from fresh human blood derived from five different healthy donors aged 25–30 years. A** &**B** denote the impact of 30 μM ebselen and **C** &**D** denote the impact of 7.5 mM NAC. The bars indicate the mean ± S.D. Values statistically different from their controls (Student’s *t*-test, 95% confidence level) are indicated with an asterisk. ** p < 0.01, *** p < 0.001 - Significantly higher than the levels of DNA damage in non-supplemented cells.

**Figure 10:**
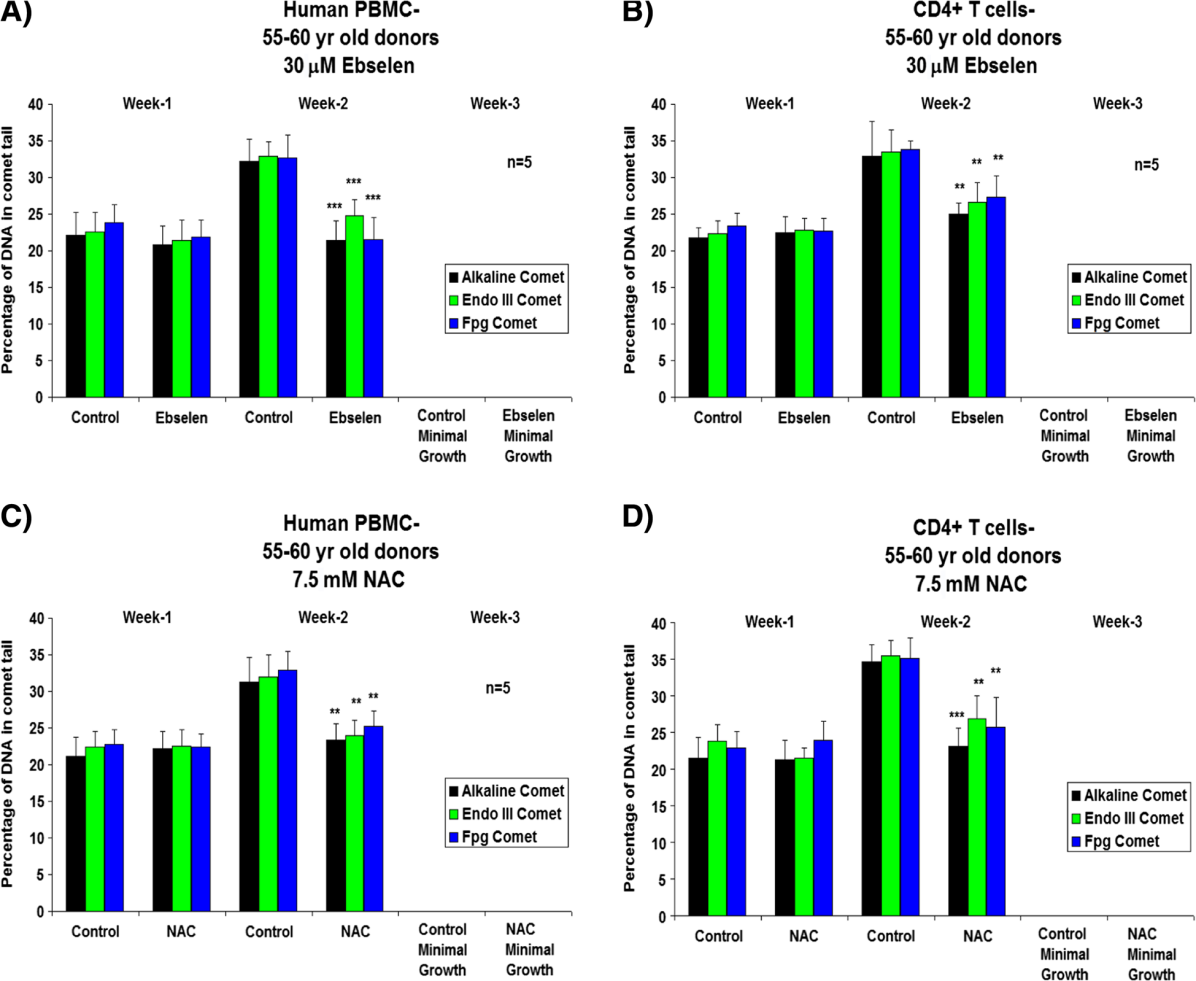
**The effect of a three week *ex vivo *supplementation with 30 μM ebselen or 7.5 mM NAC on the levels of oxidative DNA damage in human PBMCs or CD4**^**+ **^**T cells *ex vivo *isolated from fresh human blood derived from five different healthy donors aged 55–60 years. A** &**B** denote the impact of 30 μM ebselen and **C** &**D** denote the impact of 7.5 mM NAC. The bars indicate the mean ± S.D. Values statistically different from their controls (Student’s *t*-test, 95% confidence level) are indicated with an asterisk. ** p < 0.01, *** p < 0.001 - Significantly higher than the levels of DNA damage in non-supplemented cells.

Figures 9 and 10 show the results of the effect of 30 μM ebselen or 7.5 mM NAC on levels of oxidative DNA damage at different time points during a three week *ex vivo* culture period of human PBMCs and CD4^+^ T cells derived from healthy donors aged 25–30 years (Figure 9) or 55–60 years (Figure 10). The results shown in Figure 9 show a significant decrease (Student’s *t*-test, 95% confidence levels) in levels of oxidative damage to DNA in PBMCs and CD4^+^ T cells *ex vivo* isolated from donors aged 25–30 years after two weeks of supplementation with 30 μM ebselen or 7.5 mM NAC, compared to non-supplemented cells. A similar antioxidant effect was revealed after 2 weeks of supplementation of cells derived from 55–60 year old donors (Figure 10). The effect of 5 mM NAC supplementation of human PBMCs or CD4^+^ T cells *ex vivo* was very similar to 7.5 mM NAC supplementation in cell samples derived from donors of either age group (results not shown). In all cases, levels of oxidative damage to DNA increased as a function of time in culture. There were no significant differences in individual cell sample levels of oxidative DNA damage from the different donors or donor group ages.

## Discussion

In this study, the impact of the antioxidants, ebselen and NAC, on established monoclonal CD4^+^ T cell cultures and primary cultures of human PBMCs and CD4^+^ T cells has been investigated. The impact of age (*in vitro* lifespan) and/or age of cell donor on markers of T cell integrity and function (DNA damage and cell proliferation) was examined.

Supplementation of CD4^+^ TCCs, derived from a healthy 26 year old donor (400–23) and a healthy 45 year old donor (385–7) with 30 μM ebselen or 7.5 mM NAC (Table 1) from a young *in vitro* (34.5 – 31.0 Initial PD) age significantly extended their lifespan. TCCs derived from a healthy 26 year old donor and a healthy 45 year old donor supplemented with 30 μM ebselen had cumulative PDs of 56.2 and 50.8 respectively compared to PDs 44.9 and 44.4 in non-supplemented clones. 7.5 mM NAC supplementation enabled the TCCs from a healthy 26 year old donor and a healthy 45 year old donor to extend their lifespan evidenced by their cumulative PDs (56.1 and 55.8 respectively) compared to the non-supplemented clones (cumulative PDs 49.1 and 47.2).

The impact of supplementation on intracellular redox status (GSH: GSSG ratio) and total glutathione levels was determined. Intracellular redox status is an important mechanism having an invaluable role as a mediator in apoptosis in many cell systems [12]. The GSH: GSSG redox couple maintains the redox environment of the cell and GSH is abundant in the cell [13] serving as an indicator of the cellular redox environment. Redox status of the cell is hindered on oxidation of even a small amount of GSH. This oxidation results in the formation of GSSG and mixed disulfides between protein sulfhydryl groups in biological systems. It will also decrease the levels of GSH, resulting in an increase in levels of GSSG and so lowering the GSH: GSSG ratio, which has been suggested to be responsible for several human diseases [14]. Supplementation with 30 μM ebselen or 7.5 mM NAC (Figures 3 and 4, A, B) resulted in a significantly higher GSH: GSSG ratio in the CD4^+^ TCCs examined. These results suggest that in the presence of this level of ebselen, intracellular radical levels were lowered as inferred from the higher GSH: GSSG ratio. NAC is a derivative of GSH formation, explaining the higher GSH: GSSG ratio on supplementation. However, neither of the antioxidants significantly altered the total glutathione levels in either of the CD4^+^ TCCs.

The results of the analysis of DNA damage levels in TCCs at various point in their lifespan revealed, as has been previously reported [15], an *in vitro* age-related increase in DNA damage levels in non-supplemented TCCs (Figures 7 and 8). The enhanced antioxidant status of the supplemented cells, higher GSH: GSSG ratio on supplementation with 30 μM ebselen or 7.5 mM NAC, was in parallel to the significant decreases in oxidative DNA damage levels after 14 weeks of supplementation in CD4^+^ TCCs from donors of both age groups, compared to the non-supplemented clones. These results do suggest that the DNA damaging free radical/oxidant burden in the supplemented cells was less than for non-supplemented cells.

Previous findings reveal that intracellular reduced GSH levels become depleted before the onset of apoptosis [16]. The maintenance of GSH levels could explain the observed delayed time to apoptosis/cell death in the supplemented cells. This is consistent with the findings that CD4^+^ TCCs *in vitro* self-delete via apoptosis at the end of their *in vitro* lifespan.

In contrast to clones supplemented from an early point in their *in vitro* lifespan, there was no significant change to the GSH: GSSG ratio or total glutathione levels, proliferative capacity, lifespan or oxidative DNA damage levels, in TCCs derived from a 26 year old donor or a 45 year old donor on supplementation from the midpoint of their *in vitro* age (58.7 & 63.4 Initial PD) with any of the concentrations of ebselen or NAC, compared to the non-supplemented clones. These results were in line with the previous findings from our group that revealed no significant changes in longevity in CD4^+^ TCCs on long term culture, from the midpoint of their *in vitro* lifespan, with 20 mM carnosine [5]. The suggested reason for the failure of carnosine to reveal its antioxidant potential may have been due to the high background of biomolecule damage that already existed in these T cells, accumulated during earlier stages of their lifespan under conditions of 20% O_2,_ that may have decreased the antioxidant or free radical scavenging potential of carnosine. Previous findings from the group of Barnett have demonstrated an age-related increase in levels of oxidised purine and pyrimidine bases within T cells [1] which is in line with the findings reported here. Greater levels of radicals in older TCCs may affect the function of their defence systems. Published papers have reported a decrease in other markers of T cell integrity with age. The DNA repair capacity in T cells *in vitro* has been shown to decline with age, revealed in previous studies by this group [6,7]. Furthermore, Heat Shock Protein activity has been demonstrated to decline in response to age making the body more susceptible to stress-induced damage [17,18].

In PBMCs and CD4^+^ T cells *ex vivo* derived from donors from either age groups, supplementation with 30 μM ebselen or 7.5 mM NAC resulted in a significantly higher GSH: GSSG ratio (Figures 5 and 6), total glutathione levels and proliferative capacity (Figures 1 and 2) at different time points during their life in culture, compared to non-supplemented cells *ex vivo*. The levels of oxidative DNA damage were significantly decreased after two weeks of culture in human PBMCs and CD4^+^ T cells *ex vivo* (Figures 9 and 10), when compared to non-supplemented cells *ex vivo*.

The significant increase in total glutathione levels on supplementation with 7.5 mM NAC demonstrates the ability of NAC to contribute to enhanced levels of glutathione synthesis in these cells *ex vivo*, thereby enhancing the intracellular antioxidant status. A significant increase in total glutathione levels was also observed on supplementation with 30 μM ebselen. Ebselen is glutathione peroxide (GPx) mimetic. GPx has the ability to scavenge organic and inorganic peroxides and hydroxyl radicals using GSH as substrate, which in turn becomes depleted. From the results reported in this paper, ebselen may have scavenged the existing intracellular radicals, in so doing the need for GSH to scavenge them would be lessened, leading to higher GSH levels in the cells supplemented with ebselen.

The results reported in this paper provide evidence for the antioxidant potential of 30 μM ebselen or 7.5 mM NAC, against endogenously generated oxidants and those associated with the 95% air, 5% CO_2_ culture conditions in human PBMCs and CD4^+^ T cells *ex vivo*. The higher GSH: GSSG ratio represents an increased antioxidant state on supplementation with ebselen or NAC. This increase in antioxidant status helps explain the decrease in levels of oxidative DNA damage and the increase in proliferative capacity of the supplemented cells. 5 mM NAC supplementation also revealed a significant antioxidant potential, similar to supplementation with 7.5 mM NAC, in human PBMCs and CD4^+^ T cells *ex vivo*.

A comparative analysis did not reveal any differences in the effects of 30 μM ebselen or 7.5 mM NAC in human CD4^+^ T cells *ex vivo* or CD4^+^ TCCs *in vitro* in the markers of T cell integrity and function examined. Interestingly, 5 mM NAC supplementation did not reveal any significant antioxidant potential in CD4^+^ TCCs *in vitro*, unlike the potential demonstrated in the *ex vivo* polyclonal PBMC population and CD4^+^ T cells *ex vivo*. Human TCCs are maintained in culture throughout their lifespan over several months. This is not the case in cells *ex vivo*. It may be that the longer span of culture *in vitro* altered certain aspects of the biochemical/molecular status of the cells. The *in vitro* TCCs may have steadily accumulated biomolecule damage during their lifespan under conditions of 20% O_2_ that may have decreased the antioxidant or free radical scavenging potential of NAC at this specific concentration on long-term supplementation. Under these extensive culture conditions, antioxidant or free radical scavenging effects of NAC at 5 mM concentrations may be insufficient to reveal any significant antioxidant potential in TCCs *in vitro*.

The impact of long term supplementation of ebselen or NAC has not yet been reported for other cell lines. The previous studies investigating the impact of ebselen in HepG_2_[19], HL-60 [20] and glioblastoma cell lines [21] or the impact of NAC in HepG_2_[19] and HeLa [22] cell lines to name a few, revealed their potential impact only up to 24 hours after the antioxidant supplementation.

Furthermore, in this investigation we found that higher concentrations of ebselen (60-100 μM) or NAC (10 mM) resulted in complete inhibition of the growth of TCCs. It may be that higher concentrations of ebselen or NAC scavenged intracellular oxygen free radicals to such an extent that those necessary for the proliferative pathways were severely/completely limited. Other studies have demonstrated a significant increase in intracellular GSH depletion with an increase in ebselen concentration in HepG_2_ cells [19]. Ebselen has the ability to bind with GSH to form ebselen selenyl sulphide which reacts with excess of GSH to form a ebselen selenol intermediate that in turn reacts with peroxides [23]. The ebselen selenol intermediate can further react with ebselen selenyl sulphide to form ebselen diselenide. This in turn can further react with peroxides resulting in intracellular GSH depletion that leads to induction of apoptosis [19] or cells succumbing to stress [24]. This mechanism may also contribute to the complete inhibition of growth of TCCs on supplementation with higher concentrations of ebselen.

The dose- and time-dependent differences on the impact of radical scavenging capacity or pro-oxidant effect of antioxidants has been previously demonstrated [8]. Although ROS are generally thought of as harmful molecules, they play an important role in T cell signalling events such as protein tyrosine phosphorylation and activation of JNK [25]. One of the prominent families of protein kinases, the MAP kinases, includes ERK, JNK (SAPK) and P38 kinase involved in proliferation, differentiation and apoptosis in cells. The impact of ebselen or NAC on these pathways in CD4^+^ TCCs of different age group maintained at 20% O_2_ tension is currently under investigation.

## Conclusions

This current study revealed that for a limited set of conditions, 30 μM ebselen or 7.5 mM NAC supplementation of CD4^+^ TCCs (from a young *in vitro* age) or CD4^+^ T cells *ex vivo* enhanced the antioxidant potential of supplemented cells through boosting the GSH: GSSG ratio, resulting in a significant decrease in levels of oxidative DNA damage and a significant increase in lifespan, and/or proliferative capacity. It is suggested that the possible *in vivo* antioxidant potential of ebselen or NAC should be investigated alongside their potential as anti-immunosenescent interventive strategies.

## Methods

The first part of this investigation was designed to determine the impact of a range of concentrations of ebselen (0-100 μM) or NAC (0-10 mM) on the intracellular redox status and on certain markers of T cell integrity and function in a set of established CD4^+^ TCCs exposed to these agents at different timepoints in their *in vitro* lifespan. The results obtained were then used to design the second investigation on the impact of selected concentrations of ebselen or NAC on human PBMCs and in CD4^+^ T cells in primary cultures.

### T cell clones

The 400–23 clone was obtained from a 26 year old overtly healthy female laboratory worker and the 385–7 clone was obtained from a 45 year old overtly healthy female laboratory worker as described previously [18]. The TCCs, expressing αβ-T cell receptors for antigen (TCR), CD3^+^, and CD4^+^ were separately maintained in culture in a serum-free medium, X-Vivo 10 (Bio Whittaker), in 24 well plates (5 wells, 2 ml medium per well) at concentrations of 2–4 × 10^5^ cells per well, along with 2 × 10^5^ gamma-irradiated (80 Gy) RJK853 cells per well (EBV-transformed B-lymphoblastoid cell line with complete hprt deletion) as feeder cells. The TCCs were maintained in a 7-day cycle at 37°C under conditions of 5% CO_2_ and 95% air atmosphere and supplemented with 400U/ml recombinant IL-2 (Chiron, UK) on days 1 and 4 of the cycle. On day 7, a viable cell count was performed on harvested cells using a Neubauer Counting Chamber, and a new culture cycle was set up with fresh medium and RJK853 feeder cells [5,15].

The number of population doublings (PDs) that the TCCs underwent during a week in culture is referred to as the proliferative capacity of the clone. The total number of population doublings that the TCCs underwent during culture is defined as lifespan. A TCC has to expand up to 26 PD to have sufficient numbers for analysis or stock reserves, due to constraints imposed by the cloning process. The earliest stage in the lifespan of the TCCs used in this analysis was ~ 26 PD.

TCC samples, at different stages in their *in vitro* lifespan, were cryopreserved in a medium made up of 10% DMSO (Sigma), 20% FBS (Invitrogen) and 70% X-Vivo 10 and stored in liquid nitrogen for further analysis at the end of a 7 day growth cycle [5].

### Subject selection, sample collection, culture and storage of human peripheral blood mononuclear cells

This part of the investigation was granted ethical approval by Nottingham Trent University. The human blood samples were drawn from ten healthy, non-smoking males fasted overnight, five each from the age groups of 25–30 years and 55–60 years. The subjects were not on any form of medication and had not suffered any recent illness. Other lifestyle factors such as alcohol consumption and exercise regime were also taken into consideration. Human blood samples were drawn in the morning between 8.00 and 9.00 am by a licensed phlebotomist and collected in a tube containing heparin to prevent coagulation. The samples were kept on ice and taken to the laboratory within 5 minutes for separation of PBMCs.

PBMCs were isolated from fresh heparinised venous blood by density gradient centrifugation on Ficoll-Isopaque (PAA Laboratories) in LeucoSep (Lymphocyte separation tubes). PBMCs were maintained in culture separately using an established method [26]. PBMCs were washed twice in phosphate-buffered saline or X-Vivo 10 media and then maintained in culture similar to TCCs as explained in section 5.1.

### Isolation of CD4^+^ T cells from human PBMCs

The CD4^+^ T Cell Isolation Kit (Miltenyi Biotech) is a magnetic labelling system for the isolation of CD4^+^ T cells from human PBMCs. 10 × 10^6^ human PBMCs from each of the donor samples were separately treated with a cocktail of biotin-conjugated antibodies against non-CD4^+^ T cells and were subsequently depleted by labelling with anti-biotin microbeads. The different cell suspensions were then allowed to pass separately through a PBS (Oxoid Ltd) pre-rinsed column placed in the magnetic field of a suitable MACS separator. CD4^+^ T cell eluates were collected in the bottom tube. The CD4^+^ T cell eluates were then washed in phosphate buffered saline or X-Vivo 10 media and maintained in culture separately, as described in section 5.1.

### T cell proliferation assays

Human PBMCs or CD4^+^ T cells *ex vivo* from donors of different age groups, with or without the antioxidants, were cultured separately at 1 × 10^6^ cells per well in a 24 well plate. Tritiated thymidine was added 18 hours prior to harvesting at a final concentration of 0.037 MBq/ml. Cells were transferred into a 96 well plate the next day and were harvested using a 96 well harvester onto a 96 well filter plate and 40 μl of scintillation fluid was added to each of the filter pads. Filters were counted for 1 minute per well with a Top-Count scintillation counter and the amount of radioactivity incorporated (proliferation capacity of the cells) was expressed in thymidine incorporation (H^3^) counts per minute (cpm). All the samples were analysed in duplicate.

### Ebselen or NAC supplementation

The proven ability of NAC to act as an efficient antioxidant in HeLa cells [22] and HepG_2_ cells [8] was taken into consideration before choosing the dose range of the antioxidants in this investigation. Initial studies were carried out by supplementing a TCC with a range of doses of ebselen (0-100 μM; Sigma) and NAC (0-10 mM; Sigma) in X-Vivo 10. The dose ranges of 0-30 μM for ebselen and 0–7.5 mM for NAC were then chosen for the studies investigating the impact of these antioxidants on the TCC supplemented from a ‘young’ *in vitro* age. Supplementation with ebselen or NAC was on days one and four of the weekly growth cycle. The protocol for the supplementation with these antioxidants was the same as that previously used by our group for studies on the antioxidant carnosine [5].

The impact of a range of doses of ebselen or NAC on the TCC was considered before investigating the biological effect of 30 μM ebselen or 5 mM/7.5 mM NAC on human PBMCs and CD4^+^ T cells *ex vivo*.

### Assessment of the GSH: GSSG ratio and total glutathione level in human PBMCs and the TCCs

The GSH: GSSG ratio assay kit (Calbiochem) was optimised for use in TCCs on a range of cells from 0.5 × 10^6^ to 3 × 10^6^ cells/ml for assessing the levels of total glutathione and 2 × 10^6^ to 7 × 10^6^ cells/ml for assessing the levels of GSSG. A higher number of cells were used for GSSG measurement, since it is present in such low concentrations in human tissue. The same procedure was carried out for human PBMCs *ex vivo*. The cell extracts for analysis were prepared from harvested cells (2 × 10^6^ cells/ml for GSH and 5 × 10^6^ cells/ml for GSSG) according to the manufacturer’s instructions. The GSH: GSSG ratio and the total glutathione levels were calculated from the GSH and GSSG concentrations of the T cells *ex vivo* or *in vitro*, photometrically measured at 412 nM in μM concentrations. All the samples were analysed in duplicate.

### Assessment of levels of oxidative damage to DNA

The levels and types of DNA damage in T cells *ex vivo* and *in vitro*, supplemented with or without antioxidants, at different time points throughout their lifespan were assessed using modified alkaline comet assay [5]. This method has been successfully used previously to measure DNA damage in T cells [5,15].

The Comet assay is a method used to measure DNA strand breaks in eukaryotic cells. The alkaline Comet assay involves analysing stained cells embedded in agarose in a microscopic slide using a fluorescent microscope. Prior to staining, the cells are lysed with detergent and high salt resulting in nucleoids containing super-coiled loops of DNA linked to the nuclear matrix. On electrophoresis, the loops containing a break loose their super-coiling and extend towards the anode. These loops give structures similar to comets. This technique involves electrophoresis at high pH that results in structures similar to comets viewed using a fluorescent microscope. The number of DNA breaks can be assessed by quantifying the intensity of comet tail compared to the head. The alkaline Comet assay is performed to detect DNA single strand breaks and alkali-labile lesions. The two modified Comet assays also employed in this current investigation utilised either endonuclease III (Endo III) or formamidopyrimidine glycosylase (FPG) to pre-treat the cells before analysis. Endo III or FPG (both from New England Biolabs) recognise oxidatively-modified pyrimidines, or oxidatively-modified purines, respectively. The enzymes create single-strand breaks by nicking DNA at the sites of oxidatively damaged nucleotides that can be detected with the comet assay.

The Comet assays were carried out at 4°C, to reduce the amount of repair of basal/induced levels of DNA damage. 2 × 10^4^ cells embedded in 1% low melting point agarose were overlaid on to an existing 1% high melting point agarose gel in each of the frosted slides. The slides were then maintained in a high salt alkaline lysis buffer (2.5 M NaCl, 0.1 M EDTA, 0.01 M Tris, 1% (v/v) Triton X-100, pH 10) for an hour in ice. After the slides were lysed, they were equilibrated in enzyme buffer (0.04 M HEPES, 0.1 M KCl, 0.5 mM EDTA, 0.2 mg/ml BSA, pH 8.0) for three 5 minute washes prior to treatment with the enzymes Endo III or FPG. The treated slides were then incubated in a dark humid chamber at 37°C for 45 minutes. Post incubation, the slides were maintained in electrophoresis buffer (0.3 M NaOH, 1 mM EDTA) for 40 minutes, enabling the alkaline unwinding of the DNA. The slides were then electrophoresed in a pre-chilled tank at 25 V, 300 mA for 20 minutes. The slides were then neutralised with three 5 minute washes in neutralising buffer (0.4 M Tris pH 7.5). Finally, the gels were stained with 50 μg/ml ethidium bromide (Sigma) and digitally analysed using a fluorescent microscope and the comet analysis was performed on the cells detected using computer image analysis software (Komet 5.5, Andor Technology, UK), counting 50 cells per slide. The percentage of DNA damage in the comet tail was used as the quantitative measure of DNA damage. The reagents for the assay were purchased from Sigma. All the samples were analysed in duplicate.

### Statistical analysis of the samples

The results were tested for significance using paired two-sample type 2 Student’s t-tests assuming equal variances; p values are presented as appropriate.

## Abbreviations

BSA: Bovine serum albumin; CD: Cluster of differentiation; CO2: Carbon dioxide; cpm: Counts per minute; DMSO: Dimethyl sulfoxide; DNA: Deoxyribonucleic acid; EDTA: Ethylenediamine tetraacetic acid; ERK: Extracellular signal regulated kinase; GSH: Reduced glutathione; GSSG: Oxidised glutathione; Gy: Gamma irradiated; H: Tritiated thymidine; H2O2: Hydrogen peroxide; HEPES: 4-(2-hydroxyethyl)-1 piperazineethanesulfonic acid; HepG2: Hepatocellular liver carcinoma cell line; HL-60: Human promyelocytic leukemia cell line; IL: Interleukin; JNK: c-Jun N-terminal kinase; KCl: Potassium chloride; M: Molar; μM: Micromolar; mM: Millimolar; MACS: Magnetic cell separator; MAP: Mitogen activated protein; MBq: Mega becquerels; nM: Nanomolar; NAC: N-Acetyl L-cysteine; NaCl: Sodium chloride; NaOH: Sodium hydroxide; O2: Oxygen; PBMCs: Peripheral blood mononuclear cells; PBS: Phosphate buffered saline; PC-12: Pheochromocytoma cells; PD: Population doubling; ROS: Reactive oxygen species; SAPK: Stress activated protein kinase; S.D: Standard deviation; TCCs: T cell clones; TCR: T cell receptor.

## Competing interests

The authors declare that they have no competing interests.

## Authors’ contributions

SM undertook the laboratory work for this study and wrote the manuscript. YB produced the hypothesis and was PhD supervisor to SM. GP and PH gave extensive advice on the study and GP supplied the T cell clones. All authors read the manuscript, studied it critically for its intellectual content and approved the final draft.
